# Combination of dynamic transformation and dynamic recrystallization for realizing ultrafine-grained steels with superior mechanical properties

**DOI:** 10.1038/srep39127

**Published:** 2016-12-14

**Authors:** Lijia Zhao, Nokeun Park, Yanzhong Tian, Akinobu Shibata, Nobuhiro Tsuji

**Affiliations:** 1 Department of Materials Science and Engineering, Kyoto University, Yoshida-honmachi, Sakyo-ku, Kyoto 606-8501, Japan; 2 Advanced Steel Processing and Products Research Center, Department of Metallurgical and Materials Engineering, Colorado School of Mines, Golden, CO 80401, USA; 3 School of Materials Science and Engineering, Yeungnam University, Gyeongsan 712-749, Republic of Korea; 4 Elements Strategy Initiative for Structural Materials (ESISM), Kyoto University, Yoshida-honmachi, Sakyo-ku, Kyoto, 606-8501, Japan; 5 Shenyang National Laboratory for Materials Science, Institute of Metal Research, Chinese Academy of Sciences, 72 Wenhua Road, Shenyang 110016, P.R. China

## Abstract

Dynamic recrystallization (DRX) is an important grain refinement mechanism to fabricate steels with high strength and high ductility (toughness). The conventional DRX mechanism has reached the limitation of refining grains to several microns even though employing high-strain deformation. Here we show a DRX phenomenon occurring in the dynamically transformed (DT) ferrite, by which the required strain for the operation of DRX and the formation of ultrafine grains is significantly reduced. The DRX of DT ferrite shows an unconventional temperature dependence, which suggests an optimal condition for grain refinement. We further show that new strategies for ultra grain refinement can be evoked by combining DT and DRX mechanisms, based on which fully ultrafine microstructures having a mean grain size down to 0.35 microns can be obtained without high-strain deformation and exhibit superior mechanical properties. This study will open the door to achieving optimal grain refinement to nanoscale in a variety of steels requiring no high-strain deformation in practical industrial application.

Steels are basic and by far the most important materials in human civilization. Improving mechanical properties of steels, e.g., to achieve excellent balance of strength and ductility (or toughness), has always been generating great excitement in metallurgy community^1^,^2^,^3^,^4^,^5^,^6^,^7^,^8^,^9^,^10^,^11^. Among the strengthening mechanisms in steels, such as solid solution strengthening^1^, strain hardening^2^ and precipitation strengthening^9^, etc., grain refinement is the only method to improve strength and toughness simultaneously. According to Hall-Petch relationship^12^,^13^ (*σ*_*y*_ = *σ*_0_ + *k*_*y*_ · *d*^*−*1/2^, where *σ*_*y*_ is the yield stress, *σ*_0_ is the friction stress, *k*_*y*_ is a constant, and *d* is the mean grain size.), the yield strength of steels is inversely proportional to the square root of the grain diameter. Grain refinement can also result in a decrease of the ductile-to-brittle transition temperature (simplified form of Cottrell-Petch type equation: DBTT = *A* − *k*′ · *d*^−1/2^, where *A* and *k*′ are constants, and *d* is the mean grain size.), which extends the application of steels to some extreme (cryogenic) conditions^14^,^15^. Therefore, for centuries, metallurgists are endeavoring to find ways to fabricate ultrafine grained (UFG) steels (with grain size smaller than a few microns) having simple chemical compositions, which can improve the strength-to-weight ratio (specific strength) for lightweight engineering and have great benefits to replace some kinds of high-cost alloy steels with similar mechanical properties.

The control of grain refinement through thermomechanically controlled process (TMCP) where dynamic recrystallization (DRX) occurs has been extensively developed in the past decades^16^,^17^,^18^,^19^,^20^,^21^,^22^,^23^,^24^,^25^,^26^,^27^,^28^,^29^. The understanding towards DRX has been gradually deepened over time, which results in the significant development of metallurgical technology. DRX in face-centered cubic (FCC) metals and alloys was not believed to happen until the late 1970’s, when several approaches were proposed to confirm the occurrence of DRX metallographically and by the analysis of stress-strain curve^24^,^25^,^26^. The minimum grain size of austenite (FCC phase in steels) industrially achieved through DRX mechanism could be around 10 μm. For DRX of body-centered cubic (BCC) phase in steels (e.g., ferrite), it was traditionally recognized that recovery of ferrite progresses rapidly during high temperature deformation because of its high stacking fault energy and thereby the stored energy required for DRX of ferrite could not be achieved, while the theory of DRX was again reinforced by metallurgists whose work evidently confirmed the occurrence of DRX even in ferrite^22^,^27^,^28^,^29^. Nowadays, DRX has been one of the most important mechanisms to achieve grain refinement in steels. A commonly approach to grain refinement by DRX mechanism is to produce new grains directly during hot deformation. The new grains are formed from the production of recrystallization nuclei, followed by the migration of their boundaries (i.e., grain growth after nucleation)^16^,^18^,^22^. An increase in deformation temperature can enhance the formation of DRX grains, while the newly evolved grains (i.e., DRX grains) coarsen faster at a high deformation temperature^23^.

Industrially speaking, it is generally accepted that DRX of ferrite is still difficult to happen. Fully DRX ferrite structures have hardly been achieved except for the UFG interstitial free (IF) steel produced by very high strain in torsion at laboratory scale^29^. It is well established that decreasing initial grain size (prior to DRX) can enhance the DRX kinetics^30^,^31^, i.e., the formation rate of DRX grains. However, it is very difficult to enhance DRX through refining the initial grains based on the conventional techniques. Despite a tremendous expenditure of effort, the grain refinement of ferrite through DRX has encountered a limitation of several microns^20^,^21^,^32^,^33^. In order to achieve ultrafine ferrite with grain size approaching 1–2 μm, very high-strain deformation must be applied^23^,^29^,^34^,^35^,^36^, which exceeds the capacity of most of the current industrial TMCP facilities.

Another promising grain refinement mechanism for ferrite is dynamic transformation (DT)^37^,^38^,^39^,^40^,^41^,^42^,^43^,^44^,^45^, in which austenite to ferrite transformation occurs during continuous plastic deformation. Some works suggested that DRX could occur in the DT ferrite, which may lead to a further refinement of ferrite grains^41^,^42^. The study on achieving UFG steels by DT and DRX has been conducted over past decades^37^,^38^,^42^,^43^,^44^, although the actual role of DRX in enhancing ferrite grain refinement has still been under debate. In this work, we confirm the occurrence of DRX during the hot deformation of ferrite which is dynamically transformed from austenite (DT). The novelty of the DRX of DT ferrite is that the grain size of ferrite experiencing nucleation and some extent of growth (prior to DRX) could be very small. This means the required strain for the initiation of DRX and fabrication of fully ultrafine microstructure can be greatly reduced^30^,^31^. We for the first time report an unconventional temperature dependence of this DRX (of DT ferrite) behavior, which indicates an optimal condition for grain refinement. The research on the DRX of DT ferrite and its unconventional temperature dependence will reinforce the DT and DRX theory, and thus lead to new metallurgical technologies which may enable producing UFG steels with superior mechanical properties requiring no high-strain deformation in industry.

## Results

### Conventional DRX behavior (DRX of single phase)

**Figure 1:**
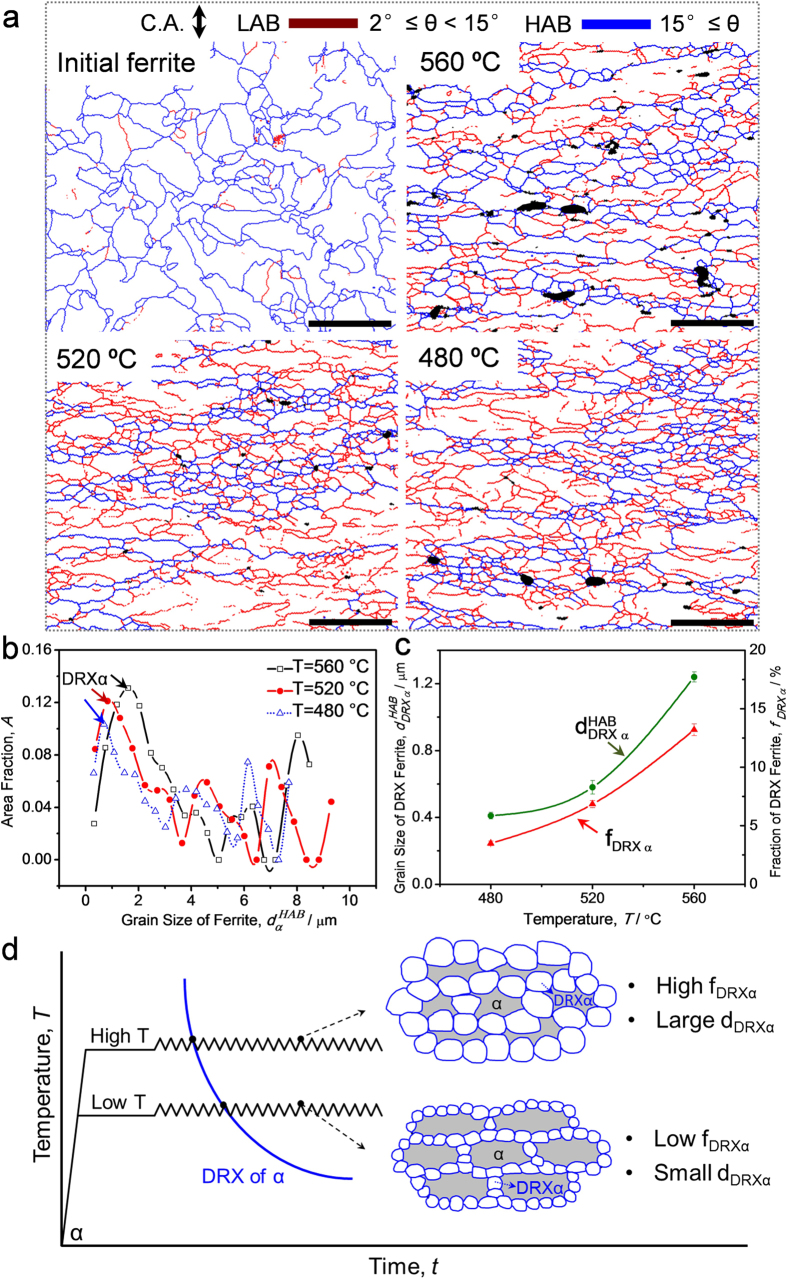
Temperature dependence of conventional DRX behavior (microstructural evolution during hot deformation of single ferrite phase). (**a**) EBSD maps showing the initial ferrite microstructure (scale bar, 20 μm) and its microstructure evolution when deformed to a strain of 0.92 at a strain rate of 10^−2^ s^−1^ at different temperatures (scale bar, 5 μm). Low-angle boundaries (LABs) with misorientation of 2–15° and high-angle boundaries (HABs) with misorientation above 15° are drawn in red and blue lines, respectively. C.A. indicates compression axis; (**b**) Distribution of grain size obtained by EBSD analysis (counting HABs) of the ferrite microstructure in (**a**); (**c**) Variations of the mean grain size (counting HABs) and volume fraction of DRX ferrite with temperature. The error bars correspond to the statistic values using EBSD maps containing 900–1000 grains at each deformation temperature; (**d**) Schematic illustration of the temperature dependence of conventional DRX behavior.

An investigation on conventional DRX behavior, especially its temperature dependence, was conducted employing a 10Ni-0.1C steel with statically transformed ferrite (fully ferrite phase with a mean grain size of 6.7 μm). Figure 1a shows EBSD grain boundary maps of the initial ferrite structure and its microstructural evolution when deformed to a strain of 0.92 at a strain rate of 10^−2^ s^−1^ at different temperatures. Low-angle boundaries (LABs) with misorientation of 2–15° and high-angle boundaries (HABs) with misorientation above 15° are drawn in red and blue lines, respectively. At 560 °C, the ferrite microstructure consists of coarse elongated grains and fine equiaxed grains. The coarse elongated grains containing LABs represent unrecrystallized microstructure during the deformation. The fine equiaxed grains surrounded by HABs are confirmed to be formed through DRX mechanism. At 520 °C, the volume fraction of DRX grains decreases, and the DRX grains become finer than those at 560 °C. With decreasing the temperature to 480 °C, less DRX grains are formed and the grain size of DRX ferrite keeps decreasing. The distribution of ferrite grain size (in diameter) obtained by EBSD analysis is shown in Fig. 1b. The multimodal distribution of grain size indicates an incomplete DRX microstructure, i.e., the one consisting of coarse deformed grains and fine DRX grains (first peaks in the curves). It is clearly indicated that the grains size of DRX ferrite increases with increasing the deformation temperature. The temperature dependence of grain size and volume fraction of DRX ferrite is illustrated in Fig. 1c. It is again confirmed that with increasing the deformation temperature, the volume fraction of DRX ferrite increases, and the grain size of DRX ferrite becomes larger.

The general view of the temperature dependence of conventional DRX behavior is illustrated in Fig. 1d. For DRX of single ferrite phase, thermal activation processes are considered important to the kinetics of DRX in the growth-controlled process^23^,^35^. The formation of DRX grains and the final grain size are dependent on Zener-Hollomon^46^ parameter *Z*:


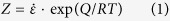


where 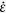, *Q, R*, and *T* refer to the strain rate, the activation energy, the gas constant, and the absolute temperature, respectively. Under the constant strain and strain rate, high temperature (low *Z*) enhances the initiation of DRX and the grain growth, resulting in higher fraction of DRX grains with larger grain size. Low temperature (high *Z*) deformation can generate more dislocations and deformation microstructures in ferrite, which could act as nucleation sites for DRX of ferrite. While due to the sluggish atom diffusion rate, low temperature leads to finer DRX ferrite with smaller volume fraction.

### DRX of dynamically transformed phase

Conventional DRX of ferrite occurs during the deformation of single ferrite phase with a certain initial grain size. Here we confirm a DRX phenomenon that can occur during the deformation of ferrite which is dynamically transformed from austenite. That is, firstly ferrite is transformed from austenite during hot deformation, then further deformed and lead to the occurrence of DRX in the continuous deformation.

**Figure 2:**
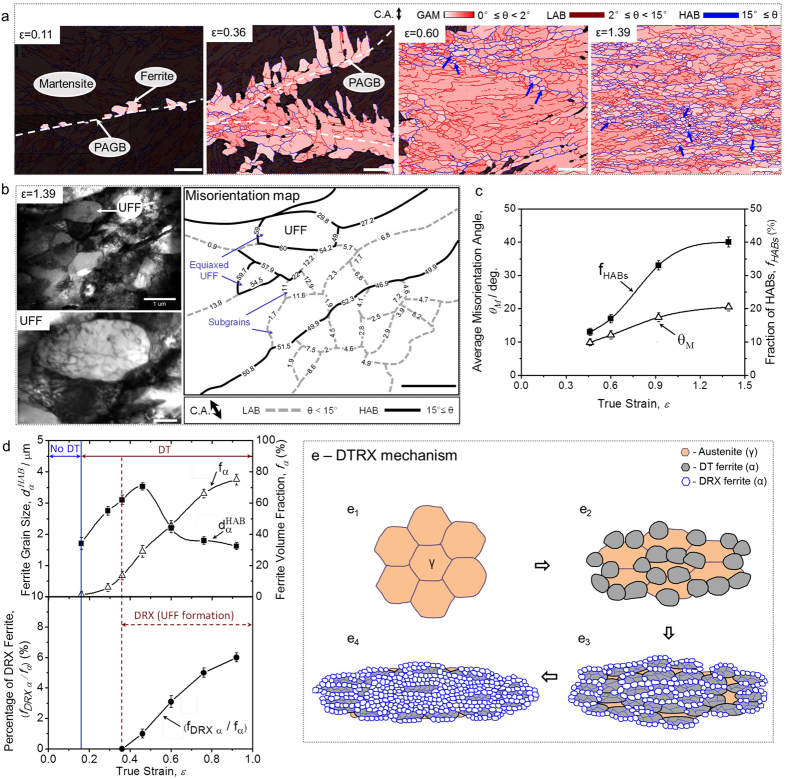
DTRX phenomenon-DRX of dynamically transformed ferrite (microstructural evolution during hot deformation of austenite). (**a**) EBSD grain average misorientation (GAM) maps of the specimens with an austenite grain size of 125 μm deformed to different strains (*ε*) at a stain rate of 10^−2^ s^−1^ at 520 °C. White broken lines indicate prior austenite grain boundaries (PAGB). Non-ferrite phases (martensite and/or austenite) were painted in black. The blue and red lines indicate high-angle grain boundaries (HABs) and low-angle grain boundaries (LABs), respectively (scale bar, 5 μm); (**b**) TEM image and corresponding misorientation map (scale bar, 1 μm) determined by Kikuchi-line analysis of the specimen deformed to a strain of 1.39. The bold black and broken grey lines indicate HABs and LABs, respectively. An enlarged area of the TEM image is marked as UFF (scale bar, 0.25 μm). C.A. indicates compression axis; (**c**) Variations of average misorientation angle and fraction of HABs with strain; (**d**) Variations of grain size (counting HABs), volume fraction of the dynamically transformed ferrite and volume fraction of the DRX ferrite with strain. The error bars in (**c**) and (**d**) correspond to the statistic values using EBSD maps containing 790–880 grains at each strain; (**e**) Schematic illustration of the DTRX mechanism.

The DRX of DT ferrite (termed as DTRX hereafter) can be interpreted by Fig. 2. Figure 2a shows EBSD grain average misorientation (GAM) maps of the specimens austenitized at 1000 °C for 300 s, cooled to 520 °C (intercritical temperature) and deformed to various strains at a strain rate of 10^−2^ s^−1^. At a strain of 0.11, very fine and nearly equiaxed ferrite grains are formed along a PAGB. With increasing the strain to 0.29, the ferrite grains grow into prior austenite grain and exhibit an irregular morphology. In addition to the coarsening of ferrite, LABs are observed within the coarse ferrite grains, indicating that a deformed structure is introduced. The fraction of LABs increases with increasing the strain to 0.60. Equiaxed ultrafine ferrite (UFF) grains surrounded by HABs start to form along the grain boundaries of coarse ferrite (as marked by blue arrows). The fraction of the equiaxed UFF increases with increasing the strain to 1.39, at which the ferrite microstructure is composed of equiaxed UFF grains and elongated coarse grains containing subgrains. The misorientation caused by deformation within each grain could be evaluated by GAM value, which can show average misorientation angle among all pairs of adjacent points in each grain. A higher GAM value (dark red color) indicates higher misorientation. The equiaxed UFF grains show lower GAM values (light red color) than the elongated coarse grains (dark red color), implying that they may be undergoing some restoration process. As shown in the TEM image and corresponding misorientation map of the specimen processed at a strain of 1.39 (Fig. 2b), equiaxed UFF grains and subgrains coexist along the grain boundaries (depicted by bold black lines) of the elongated coarse ferrite. The equiaxed UFF contains a certain amount of dislocations and has the similar grain size to the subgrains. The only difference is that the equiaxed UFF grains are surrounded by complete HABs but the subgrains are surrounded by either complete LABs (with misorientation from 0.9° to 13.9°) or partial LAB and HAB. It is already confirmed that the DT ferrite is furthermore deformed during growth and contains subgrains (Fig. 2a). Higher concentration of deformation (or inhomogeneous deformation) would occur near grain boundaries (i.e., HABs) of ferrite^47^, and could increase the misorientation angle of the subgrains and thus enhance the formation of HABs (Fig. 2c). Finally, equiaxed UFF (i.e. DRX ferrite, Fig. 2b) surrounded by HABs are formed.

The mechanism of DTRX is interpreted by Fig. 2d and e. Basically there are two regions in Fig. 2d: a region prior to the onset of DT (No-DT region, corresponding to Fig. 2e1) and a region after the onset of DT (DT-region, corresponding to Fig. 2e2–e4). In the DT-region, there is another stage where DRX occurs (UFF formation). At the beginning of DTRX, ferrite grains are transformed from austenite during deformation (Fig. 2e3). The grain size of DT ferrite firstly increases (black solid rectangle in Fig. 2d), indicating a grain growth process at a relatively early stage of transformation, but then decreases after a certain strain, which is totally different from the conventional grain growth behavior in static ferrite transformation^48^,^49^. The decrease in the ferrite grain size accords well with the development of DRX grains (black solid circle in Fig. 2d,e_3_ and e_4_) in the continuous deformation, which implies a new grain refinement process happening during deformation. Here it could be concluded that the DRX of ferrite occurs at a certain strain after the onset of DT. It is the formation of DRX grains that leads to the further grain refinement of ferrite.

**Figure 3:**
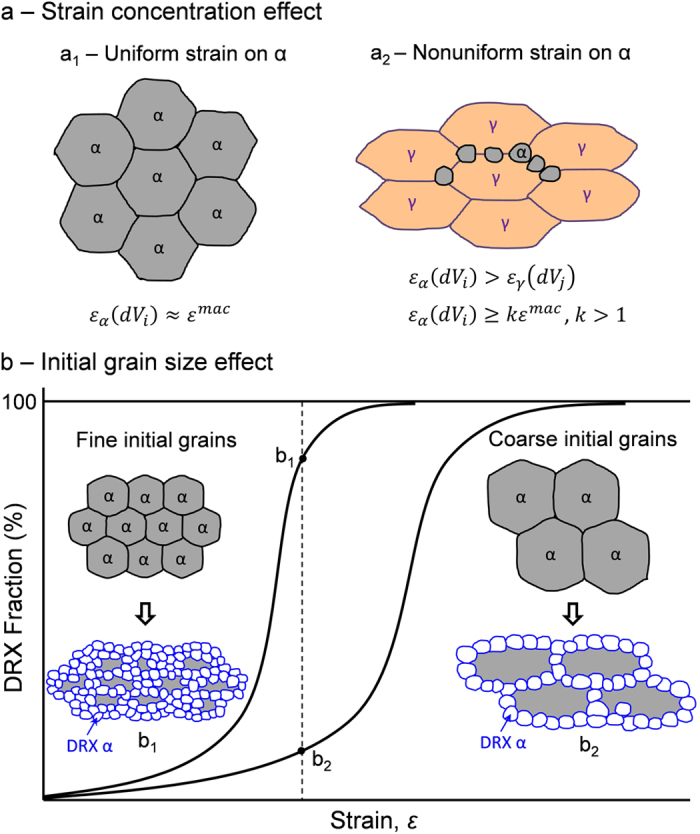
Explanation for the occurrence of DTRX phenomenon. (**a**) Strain concentration effect. (**a**_**1**_) Uniform strain on full ferrite (α) phase and (**a**_**2**_) nonuniform strain on coexisting austenite (γ) and ferrite (α). *ε*^*mac*^, *ε*_*α*_*d(V*_*i*_), *ε*_*γ*_*d(V*_*j*_) represent applied macroscopic strain, local strain on ferrite and austenite, respectively. *k* is strain concentration constant; (**b**) Initial grain size effect. (**b**_**1**_) and (**b**_**2**_) indicate the DRX microstructures obtained under the same strain from initial fine ferrite and coarse ferrite grains, respectively.

There are mainly two reasons for the occurrence of DRX of DT ferrite (DTRX). One is due to the strain concentration effect (Fig. 3a). In the case of conventional DRX of single ferrite phase (Fig. 3a1), the initial grain size of ferrite prior to deformation is constant. Theoretically, the applied macroscopic strain *ε*^*mac*^ on the bulk specimens is homogenously distributed in each ferrite grain (*ε*_*α*_*d(V*_*i*_)), i.e., *ε*_*α*_*d(V*_*i*_) ≈ *ε*^*mac*^. While in the case of the DRX of DT ferrite (Fig. 3a2), austenite is firstly deformed prior to the formation of ferrite. At high temperatures, ferrite phase is softer than the work-hardened austenite phase^30^,^50^. Once ferrite is dynamically transformed from austenite, the strain will be more concentrated on the soft ferrite phase (i.e., *ε*_*α*_*d(V*_*i*_) > *ε*_*γ*_*d(V*_*j*_)). The soft ferrite grains could absorb plastic deformation to a greater extent than the macroscopic plastic strain^51^ (i.e., *ε*_*α*_*d(V*_*i*_) ≥ *kε*^*mac*^, *k* > 1), which increases the driving force for the initiation of DRX. The other reason is attributed to the grain size effect on DRX (Fig. 3b). Finer grains have larger fraction of grain boundaries, which are the preferential nucleation sites for DRX. The smaller grains size can also enhance work-hardening behavior, which could affect the generation of dislocations and their interaction with grain boundaries and finally promote the DRX (Fig. 3b1 and b_2_). Therefore, it is believed that the decrease in ferrite grain size can not only decrease the critical strain for the operation of the DRX of ferrite, but also accelerate the kinetics of DRX^30^,^31^. In the case of DTRX, ferrite grains are dynamically nucleated and experience certain growth, which means the grain size of ferrite prior to DRX could be very small. This will significantly enhance the occurrence of DRX and accelerate the kinetics of ultrafine grains formation.

### Unconventional temperature dependence of the DTRX phenomenon

**Figure 4:**
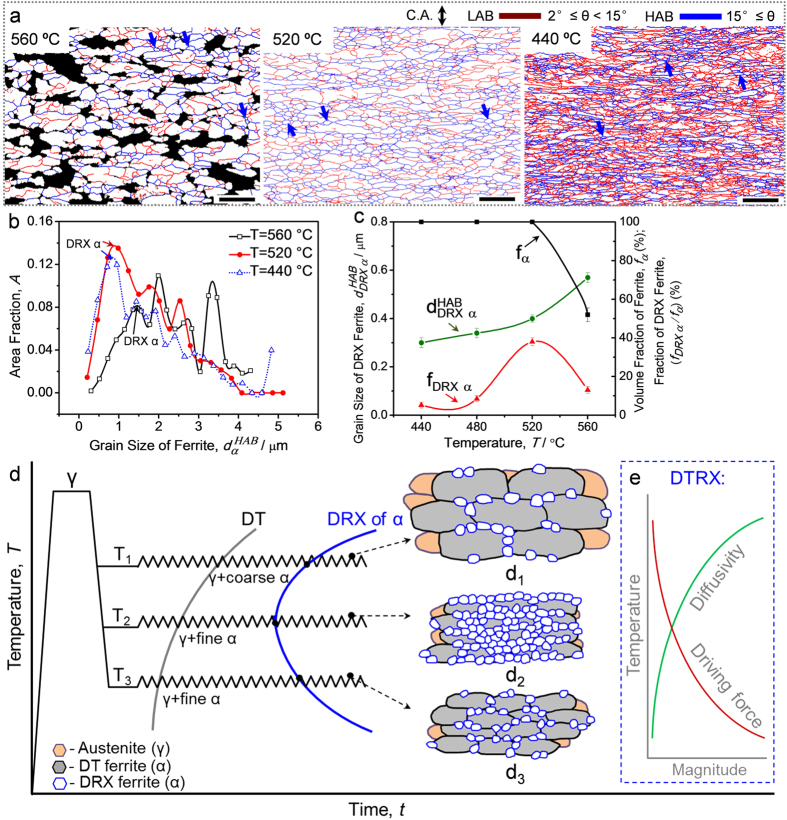
Unconventional Temperature dependence of the DTRX phenomenon. (**a**) EBSD grain boundary maps of the specimens with an austenite grain size of 10 μm deformed to a strain of 0.92 at a strain rate of 10^−2^ s^−1^ at different temperatures (scale bar, 5 μm). Non-ferrite phases were covered in black. The blue and red lines indicate HABs and LABs, respectively. C.A. indicates compression axis; (**b**) Distribution of grain size obtained by EBSD analysis (counting HABs) of the ferrite in (**a**); (**c**) Variations of the mean grain size (counting HABs, 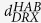), volume fraction (*f*_*DRX α*_) of DRX grains and volume fraction of dynamically transformed (DT) ferrite (*f*_*α*_) with temperature. The error bars correspond to the statistic values using EBSD maps containing 950–1100 grains at each temperature; (**d**) Schematic illustration on the unconventional temperature dependence of the DTRX behavior. (**d**_**1**_), (**d**_**2**_) and (**d**_**3**_) illustrate the microstructures obtained at the temperatures of T_1_, T_2_ and T_3_, respectively; (**e**) Variations of diffusivity of atoms and driving force for DRX of DT ferrite with temperature.

The temperature dependence of DTRX behavior is further investigated in order to compare with the conventional DRX under the same deformation condition. Figure 4a shows EBSD grain boundary maps of the DTRX specimens austenitized at 700 °C for 900 s and deformed to a strain of 0.92 at a strain rate of 10^−2^ s^−1^ at different temperatures. At 560 °C, the ferrite mostly shows coarse and elongated morphologies. The volume fraction of DT ferrite is 52% at this temperature, and only a few of DRX grains (i.e., equiaxed UFF grains surrounded by HABs) can be observed, as marked by blue arrows. At 520 °C, the fraction of DRX grains increases compared to that at 560 °C. With decreasing the temperature to 440 °C, the fraction of DRX grains (as marked by blue arrows) decreases. Most of the grains are elongated and contain a large amount of LABs, exhibiting a more deformed structure. The distributions of the ferrite grain size are shown in Fig. 4b. First peaks of the multimodal curves reflect the characteristics of the DRX grains. It is shown that with decreasing the temperature, the grain size of DRX ferrite becomes finer. It is noteworthy that the HABs surrounding ferrite grains at 440 °C in Fig. 4a are more like geometrically necessary boundaries (GNB)^52^, which are formed due to the deformation at relatively low temperatures rather than the DRX mechanism. Figure 4c summarizes the changes in grain size, volume fraction of DT ferrite and volume fraction of DRX ferrite as a function of deformation temperatures. At the temperatures from 440 °C to 520 °C, the ferrite transformation (solid rectangles) is completed after a strain of 0.92. The grain size (solid circles) of DRX ferrite increases from 440 °C to 560 °C. It is rather interesting that the fraction of DRX ferrite (solid triangles) exhibits a peak value at 520 °C, which is totally different from conventional DRX showing monotonous temperature dependence (Fig. 1c).

The reasons for the unconventional temperature dependence of DTRX behavior are interpreted by Fig. 4d and e. As shown in Fig. 4d, it has been known that lowering deformation temperature (above nose temperature in kinetic C-curve) shortens the incubation time for the onset of DT and accelerates its kinetics^36^,^53^. At a high temperature T_1_ (e.g., 560 °C), the driving force and nucleation density for ferrite transformation are low, but the diffusivity of atoms is high. Therefore, the grain growth of DT ferrite is rather dominant, resulting in a large grain size (Fig. 4a, 560 °C). The high temperature (560 °C) would enhance DRX behavior, but the large grain size retards the nucleation probability and the enhanced recovery at high temperature decreases the driving force (Fig. 4e) for the subsequent DRX. Therefore, the combined effect of the grain size and temperature is to retard the onset of DRX at a high temperature T_1_ (Fig. 4d1), resulting in the lower fraction of ultrafine ferrite grains that was experimentally observed. At a lower temperature T_2_ (e.g., 520 °C), both the driving force and the nucleation density for ferrite transformation increase, which lead to faster transformation kinetics. The fast transformation kinetics results in finer ferrite grain size due to the sufficient impingement effect between ferrite grains. Furthermore, the strain concentration on ferrite becomes higher at fast transformation kinetics. The high strain concentration and fine ferrite grain size greatly increase the driving force for DRX (Fig. 4e), thus enhance the initiation of DRX and accelerate its kinetics (Fig. 4d2). However, when the temperature is further decreased to a very low value T_3_ (e.g., 440 °C), the fraction of DRX grains becomes smaller (Fig. 4c). The transformation kinetics is surely faster at 440 °C than that at 520 °C (shown by TTT diagram in Fig. S1 in the Supplementary document), but the diffusivity of atoms is so low (Fig. 4e) that the thermal activation of DRX is suppressed. Even though the ferrite is still deformed after transformation, the deformation would introduce more GNB (Fig. 4a, 440 °C) rather than enhance DRX. The DRX behavior of DT ferrite exhibits a C-curve according to the deformation temperature, which implies an optimized temperature for the progress of DRX, to be more specific, for the grain refinement of ferrite. This will open the door to achieving optimal grain refinement of ferrite in TMCP requiring no high-strain deformation.

## Discussion

It has been well known that finer initial grains can enhance DRX and lead to finer DRX grains^30^,^31^. As a simplified description, for hot compression deformation in the present study, the grain size (*d*) counting only HABs along compression direction can be expressed as^30^


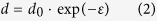


where *ε* and *d*_0_ are applied strain and initial grain size, respectively. It is indicated that if the initial grain size (*d*_0_) could be decreased, then the required strain (*ε*) to achieve DRX grains with a certain grain size would be reduced. For conventional DRX of ferrite, the initial grain size is constant. The grain refinement effect of conventional DRX is limited because it is technically difficult to enhance DRX through refining the initial grains. While for the DTRX mechanism, transformed ferrite firstly experiences nucleation and growth to some extent, which means the ferrite grain size is variable prior to DRX. Our study has proved two important points: (1) DT can greatly affect the subsequent DRX behavior; (2) There exists an optimal deformation temperature at which the kinetics of DRX of DT ferrite is the fastest. These two points imply a new grain refinement strategy by controlling two mechanisms, i.e. DT and DRX, in one TMCP route. That is, at the optimal temperature, DRX should be enhanced by accelerating DT kinetics and consequently leads to finer grains with improved mechanical properties.

**Figure 5:**
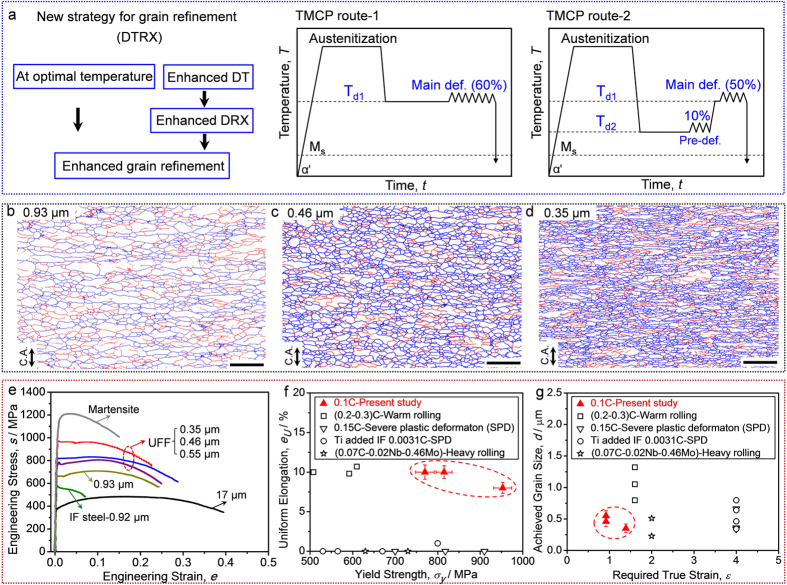
New strategy for grain refinement and mechanical properties of the ultrafine ferrite structures. (**a**) Schematic illustration on the new strategy for grain refinement; (**b**) and (**c**,**d**) are EBSD maps of the ferrite structures obtained by TMCP route-1 and route-2, respectively (scale bar, 5 μm). Low-angle boundaries (LABs) with misorientation of 2–15° and high-angle boundaries (HABs) with misorientation above 15° are drawn in red and blue lines, respectively; (**e**) Mechanical properties of the specimens processed in the TMCP route-1 (0.93 μm) and route-2 (marked by red broken circle), the specimen with coarse ferrite grains (17 μm), the specimen with full martensite of the present 10Ni-0.1C steel, and an UFG IF steel (0.92 μm) obtained by ARB (accumulative roll bonding) and subsequent annealing^54^; (**f**) Comparison of the mechanical properties between the UFF structures obtained in the present study (marked by red broken circles) and the UFF structures reported in previous works^34^,^36^,^56^,^57^. The error bars correspond to the statistic values of 3–5 tensile specimens under each tensile test condition. (**g**) Comparison between the present study and previous works^34^,^36^,^56^,^57^ on the required strain for achieving UFF structures. The error bars correspond to the statistic values using EBSD maps containing more than 1600 grains for calculating the average grain size.

Figure 5a schematically illustrates the new strategy for grain refinement based on DTRX. TMCP route-1 is just for comparison, while the route-2 illustrates the new strategy. The novelty of the TMCP route-2 is that at a relatively low temperature (T_d2_), a small pre-deformation is applied for accelerating DT and subsequent DRX at the high temperature (T_d1_). Even though the total strain is the same in the route-1 and route-2, the final microstructures are significantly different. Figure 5b and c are EBSD grain boundary maps of the ferrite structures obtained by a 60% reduction (an equivalent strain of 0.92) in TMCP route-1 and route-2, respectively. The fraction of high-angle boundaries (HABs) is 56% and the mean grain size (counting only HABs) of ferrite obtained in the TMCP route-1 is 0.93 μm. In the TMCP route-2, the fraction of HABs significantly increases from 56% to 75% (Fig. 5c), indicating that DRX of transformed ferrite progressed much more than that in the TMCP route-1. Accordingly, ferrite grains are significantly refined to 0.46 μm. While this is not the limitation of grain refinement based on the DTRX mechanism. Figure 5d is an example for showing the possibility of further grain refinement through a modified TMCP route-2 with higher deformation by 75% reduction (an equivalent strain of 1.39). The fraction of HABs is 70% and the mean grain size is 0.35 μm. It should be emphasized that it is the first time to date to obtain such an equiaxed and uniform ferrite structure with ultrafine grain size (Fig. 5c and d) in low-C steels without high strains (only 0.92 for 0.46 μm and 1.39 for 0.35 μm).

Figure 5e shows engineering stress-strain curves of the specimens processed in the TMCP route-1 and route-2 (marked as UFF), the specimen with a statically transformed coarse ferrite grains (grain size of 17 μm), the specimen with full martensite of the present 10Ni-0.1C steel, and an UFG IF steel (0.92 μm) obtained by ARB (accumulative roll bonding) and subsequent annealing^54^. Compared to the specimen with statically transformed coarse ferrite, the TMCP (route-2) processed UFF specimens (0.55 μm–0.35 μm) showed the significantly higher yield strength of 770–953 MPa and tensile strength of 810–973 MPa, which are much superior to the UFG IF steel and even close to the yield strength of martensite (1100 MPa). It has been known that UFF ferrite usually show limited uniform elongation due to early plastic instability^55^. In such a context, it is rather surprising that the present UFF obtained through DTRX mechanism maintains large tensile ductility, i.e., uniform elongation of 8–10% and total elongation of 23–29%. Compared to the results reported in literatures^34^,^36^,^56^,^57^ (Fig. 5f), the plots of uniform elongation and yield strength of the present UFF structures are out of the trend in UFF steels obtained through processes including heavy plastic deformations. And importantly, it requires so far the smallest strain to achieve UFF structure using the DTRX mechanism (Fig. 5g). The present TMCP routes for producing UFF structures require a relatively low Ms temperature of the steel. Therefore, for industrial application of the present TMCP routes to achieve ultrafine grained steels, some alloying elements should be added in the steels to decrease the Ms temperature^58^,^59^. It is our hope that the DTRX, and its unconventional temperature dependence, may broaden the opportunity for the fabrication of ultrafine grained steels with superior mechanical properties in TMCP without high-strain deformations.

## Methods

### Materials preparation

The material used in this study is a 10Ni-0.1C steel, of which the chemical composition and temperature-time-transformation (TTT) diagram are shown in Supplementary Table S1 and Fig. S1. The Ortho-equilibrium temperature (Ae_3_) and para-equilibrium temperature (Ap_3_) of the steel, calculated by Thermo-Calc software, are 686 °C and 583 °C, respectively. The as-received hot-rolled plate 15 mm thick was cold-rolled to a thickness of 11.2 mm with a reduction of 25%, and then homogenized at 1100 °C for 172,800 s in vacuum. Two procedures were designed for the sample preparation. In the first case (specimens for new DTRX study), the homogenized plate was water-quenched after the homogenization at 1100 °C to have a martensite structure. In the other case (specimens for conventional DRX study), the homogenized plate was firstly water-quenched to obtain martensite, then austenitized again at 750 °C for 900 s or at 900 °C for 1,800 s, and slowly furnace-cooled to obtain a fully ferrite microstructure with a mean grain size of 6.7 μm and 17 μm, respectively. Cylindrical specimens with a height of 12 mm and a diameter of 8 mm were machined from the two kinds of plate and installed in a thermomechanical processing simulator (Thermecmastor-Z, Fuji Electronic Industrial Co. Ltd.) for conducting thermomechanically controlled processes (TMCP).

### Thermomechanically controlled processes

TMCP including compression deformations were conducted at high temperatures achieved by induction heating in a vacuum chamber. For the hot deformation of single ferrite structure (conventional DRX of ferrite), the specimens with a mean grain size of 6.7 μm were heated from room temperature to the deformation temperatures from 480 °C to 560 °C at a rate of 10 °C s^−1^, held for 60 s to homogenize the temperature, and then uniaxially compressed to a strain of 0.92 at a strain rate of 10^−2^ s^−1^ followed by water-quenching.

For the hot deformation of austenite (DRX of dynamically transformed ferrite, i.e. DTRX), the specimens were austenitized at 1000 °C for 300 s or at 700 °C for 900 s to obtain different austenite grain sizes of around 125 μm or 10 μm, respectively. The austenitized specimens were cooled to the deformation temperatures ranging from 560 °C to 440 °C at a rate of 30 °C s^−1^, held for 60 s to homogenize the temperature of the cylindrical specimens, and then uniaxially compressed to various strains ranging from 0.05 to 1.39 (5% to 75% reduction in height) at a strain rate of 10^−2^ s^−1^ followed by water-quenching.

For the study of new strategies for grain refinement, two kinds of TMCP routes were designed. The TMCP route-1 is for comparison, the TMCP route-2 is newly designed for obtaining ultrafine ferrite with grain sizes of 0.55 μm, 0.46 μm (Fig. 5c) to 0.35 μm (Fig. 5d). The austenitizing condition is the same in the two routes. In the TMCP route-1, the specimens were austenitized at 700 °C for 900 s, cooled to 520 °C at a rate of 30 °C s^−1^, held for 60 s to homogenize the temperature, and then uniaxially compressed by 60% reduction (logarithmic equivalent strain of 0.92) at a strain rate of 10^−2^ s^−1^ followed by water quenching. The TMCP route-2 is composed of two-step deformation at different temperatures. The austenitized specimens were firstly cooled to 410–300 °C (above the Ms temperature (250 °C)) at a rate of 30 °C s^−1^, deformed by 10% reduction (or 30% reduction for the case of 0.35 μm) at a strain rate of 10^0^ s^−1^, reheated up to 520 °C at a rate of 55 °C s^−1^ and then deformed by 50% reduction (45% reduction for 0.35 μm) at a strain rate of 10^−2^ s^−1^ (10^−1^ s^−1^ for 0.35 μm) followed by water quenching.

### Microstructure characterization

Microstructures at the center of the cylindrical specimens on the sections parallel to the compression axis were characterized by a field-emission type scanning electr on microscope (FE-SEM, FEI XL30S FEG) equipped with an electron back-scattering diffraction (EBSD) system operated at an accelerating voltage of 15 kV and a transmission electron microscope (TEM, Philips CM200FEG) at 200 kV. For the EBSD measurement, the specimens were mechanically polished using 4000 grit SiC paper and then electrically polished in a solution of 10% HClO_4_ and 90% CH_3_COOH at 20 °C. For the TEM observations, thin-foil specimens were prepared by twin-jet electropolishing using the same solution as that for EBSD.

### Miniature tensile tests

Sheet-type specimens for tensile test were machined from the compressed samples. The tensile direction was perpendicular to the compression direction. Small-size tensile specimens with gage length, width and thickness of 2 mm, 1 mm and 0.5 mm, respectively, were cut from the compressed specimens, so that the gage parts corresponded to the center of the specimens. A CCD camera taking 10 images per second was used to precisely measure the displacement during the tensile test. Tensile test was conducted at an initial strain rate of 8.3 × 10^−4^ s^−1^ at room temperature. The present study confirmed the reliability of the small-size tensile specimens by comparing the engineering stress-strain curves of the small-size and large-size (gauge length-10.0 mm, width-5.0 mm, thickness-1.0 mm) tensile specimens cut from the same 10Ni-0.1C steel with full ferrite phase (grain size of 6.7 μm), as shown in the Supplementary Fig. S2.

## Additional Information

**How to cite this article:** Zhao, L. *et al*. Combination of dynamic transformation and dynamic recrystallization for realizing ultrafine-grained steels with superior mechanical properties. *Sci. Rep.* **6**, 39127; doi: 10.1038/srep39127 (2016).

**Publisher's note:** Springer Nature remains neutral with regard to jurisdictional claims in published maps and institutional affiliations.

## Supplementary Material

Supplementary Information
