# Supplementary material for: Combination of dynamic transformation and dynamic recrystallization for realizing ultrafine-grained steels with superior mechanical properties
Source: Sci Rep. 2016 Dec 14;6:39127. doi: 10.1038/srep39127 (PMC5155429; doi:10.1038/srep39127)
Supplement: Supplementary Information [file srep39127-s1.pdf]

Combination of dynamic transformation and dynamic recrystallization for realizing ultrafine-grained steels with superior mechanical properties

Lijia Zhao [1,2]*, Nokeun Park [1,4], Yanzhong Tian [1,3,5], Akinobu Shibata [1,3], Nobuhiro Tsuji [1,3]*

[1] Department of Materials Science and Engineering, Kyoto University, Yoshida-honmachi, Sakyo-ku, Kyoto 606-8501, Japan

[2] Advanced Steel Processing and Products Research Center, Department of Metallurgical and Materials Engineering, Colorado School of Mines, Golden, CO 80401, USA

[3] Elements Strategy Initiative for Structural Materials (ESISM), Kyoto University, Yoshida-honmachi, Sakyo-ku, Kyoto, 606-8501, Japan

[4] School of Materials Science and Engineering, Yeungnam University, Gyeongsan 712-749, Republic of Korea

[5] Shenyang National Laboratory for Materials Science, Institute of Metal Research, Chinese Academy of Sciences, 72 Wenhua Road, Shenyang 110016, P.R. China

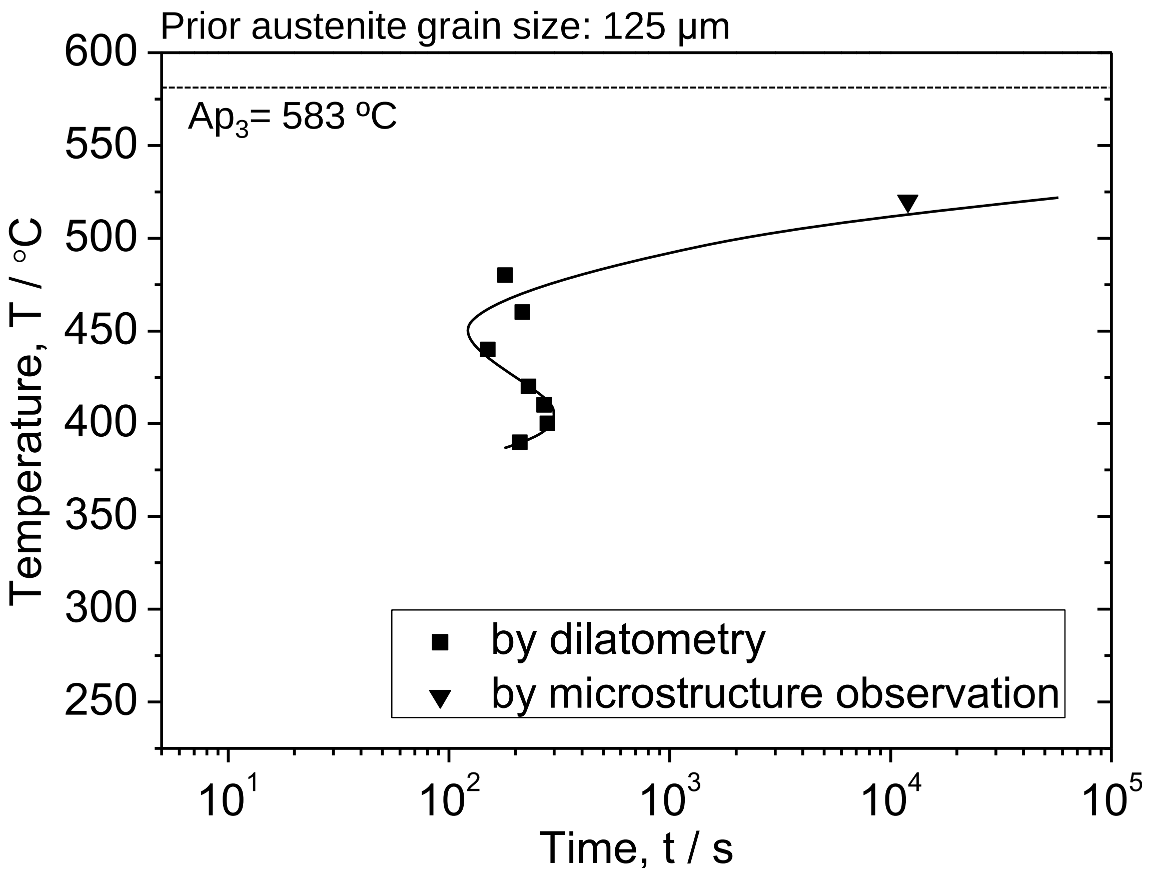

Supplementary Fig. S1 Time-temperature-transformation (TTT) diagram of the 10Ni-0.1C steel.

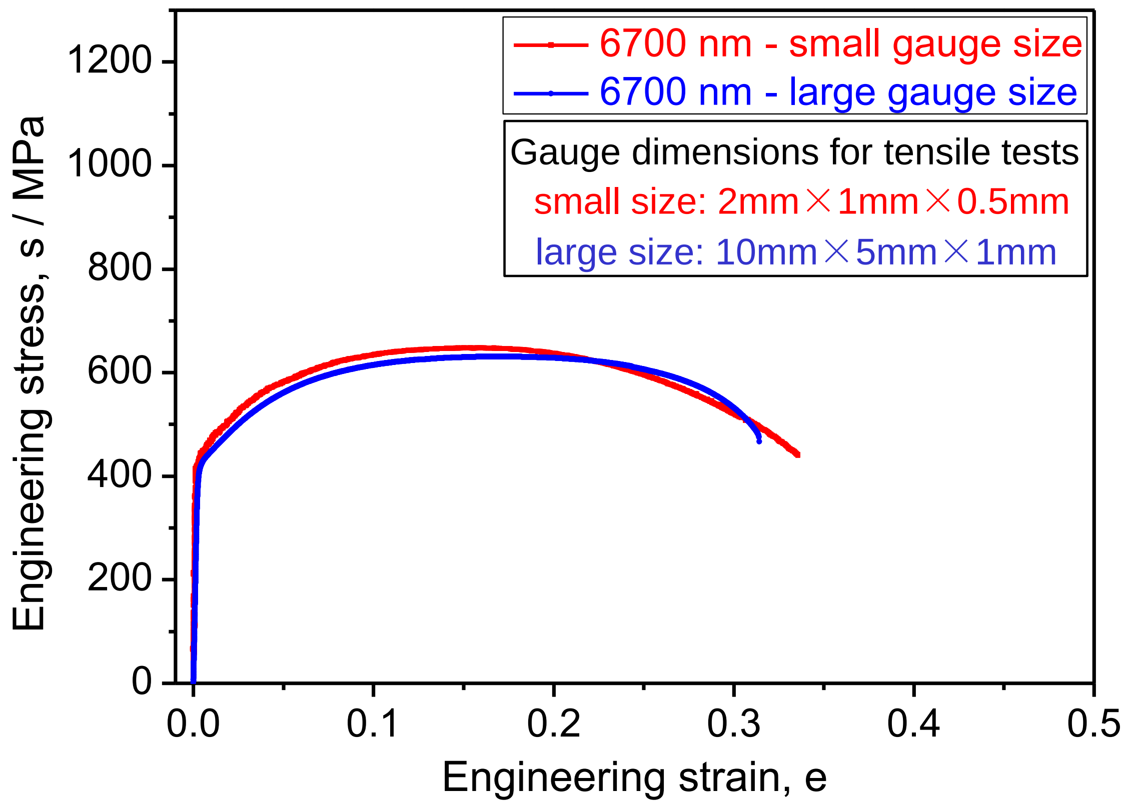

Supplementary Fig. S2 Confirmation of reliability of the mechanical test-Engineering stress-strain curves of the specimens with coarse grained (6700 nm) ferrite (Fe-10Ni-0.1C alloy) having small and large gauge sizes. Small-size and large-size tensile specimens were used to confirm the reliability of the small-size specimen.

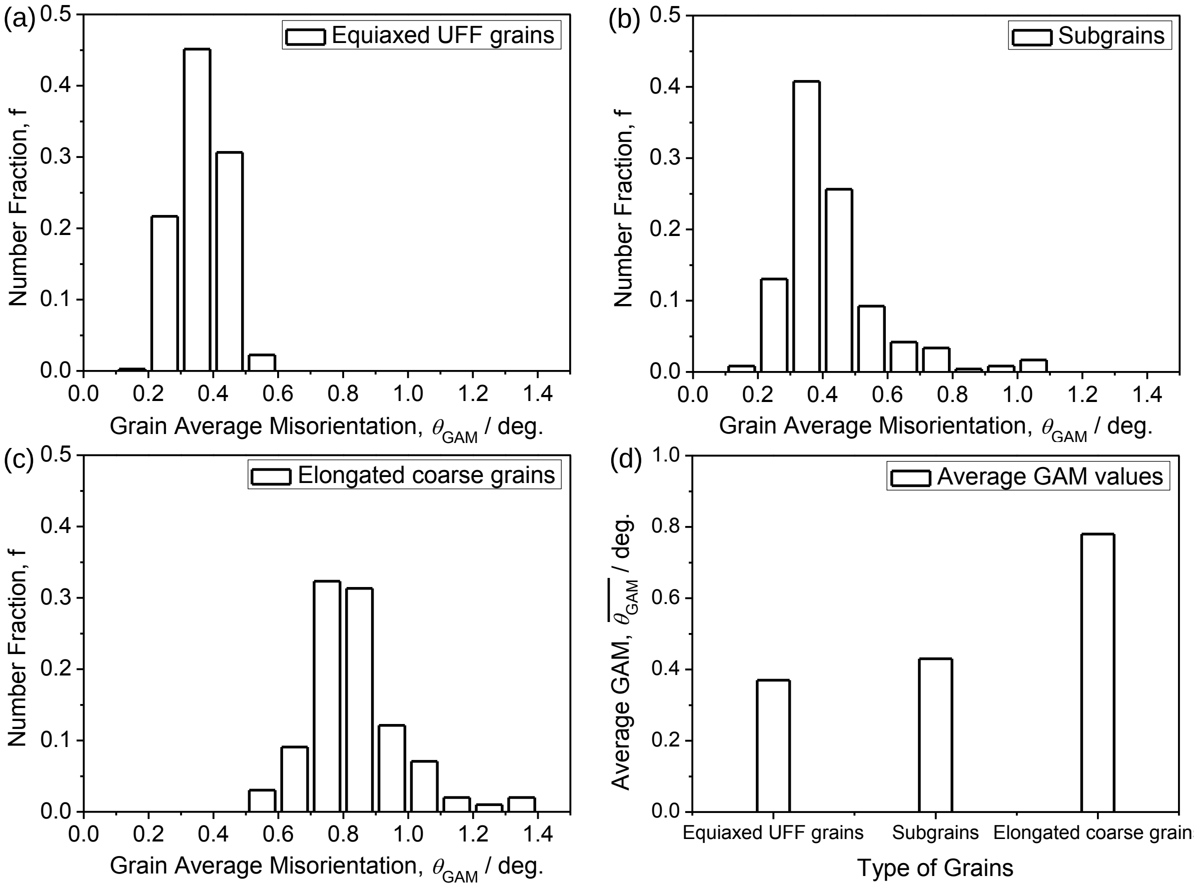

Supplementary Fig. S3 Comparison of misorientation in different types of grains- Quantitative analysis of the distribution of GAM values of (a) equiaxed UFF grains, (b) subgrains and (c) elongated coarse grains in **Fig. 2a** of the paper. (d) Average GAM values of the three types of grains.

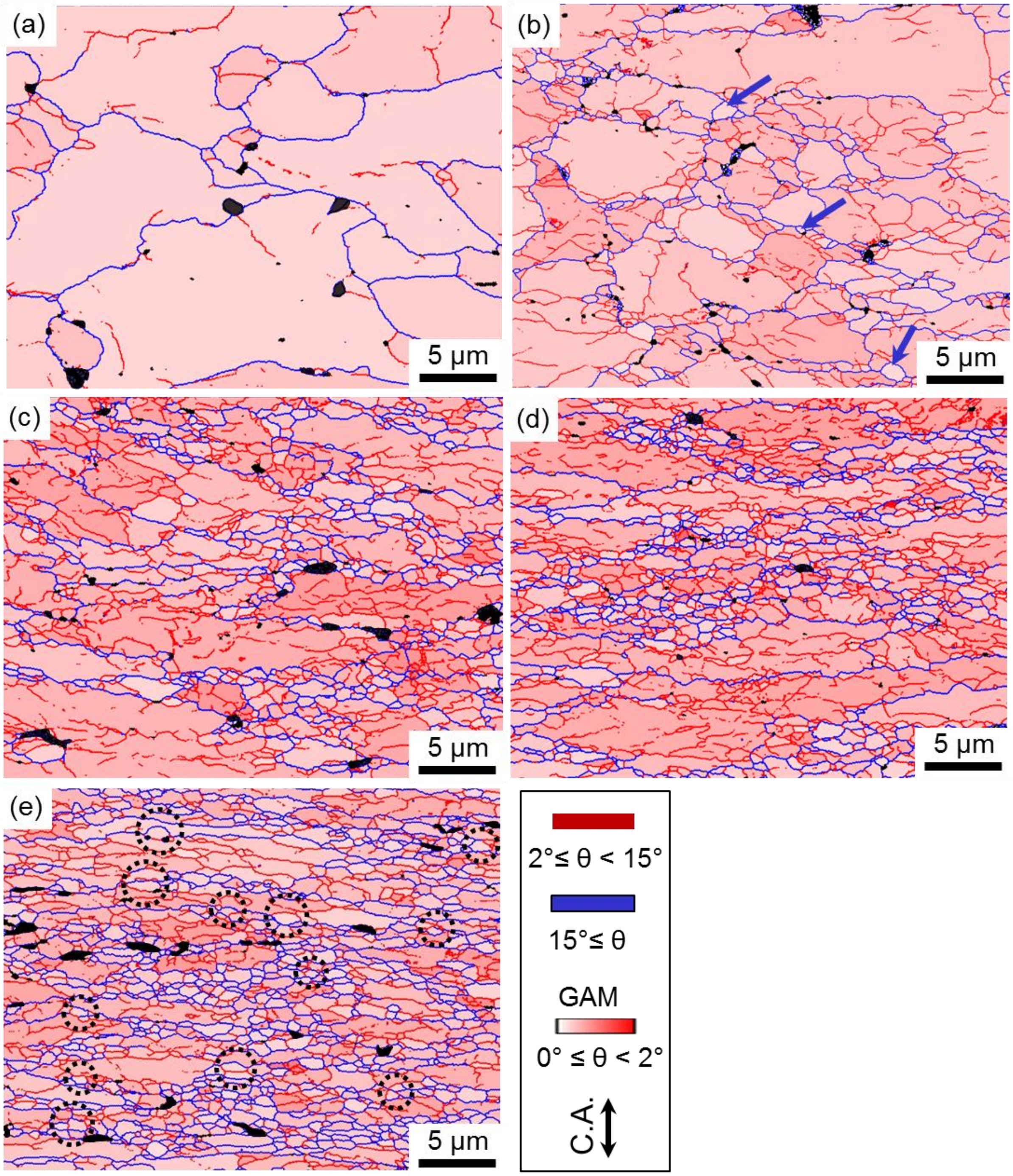

Supplementary Fig. S4 Occurrence of DRX during hot deformation of single ferrite phase- Grain average misorientation (GAM) maps of the specimens with an initial ferrite grain size of 6.7 µm deformed to a strain of (a) 0.16, (b) 0.36, (c) 0.60, (d) 0.92 and (e) 1.39 at 10^{-2} s^{-1} and 520°C. Non-ferrite phase (i.e., reversely transformed austenite during deformation of ferrite) was painted in black. Low-angle boundaries with misorientation of 2-15° and high-angle boundaries with misorientation above 15° are drawn in red and blue lines, respectively. Blue arrows in (b) and black dotted circles in (e) mark equiaxed UFF grains and subgrains, respectively. Compression axis is along the vertical direction of the maps.

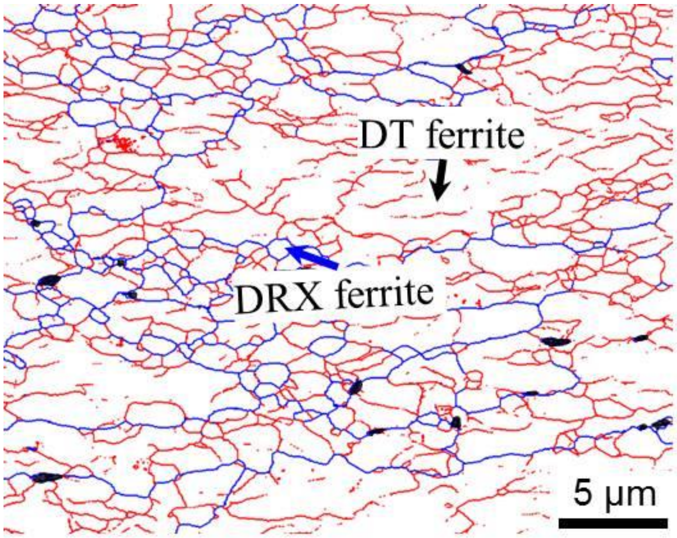

Supplementary Fig. S5 Microstructure consisting coarse dynamically transformed (DT) ferrite grains and fine dynamically recrystallized (DRX) ferrite grains-EBSD grain boundary map of the austenitized specimen deformed to a strain of 0.92 at a strain rate of 10^{-3} s^{-1} and 520°C. Non-ferrite phase was painted in black. Low-angle boundaries (LABs) with misorientation of 2-15° and high-angle boundaries (HABs) with misorientation above 15° are drawn in red and blue lines, respectively. Blue arrow and black arrow point out DRX ferrite and DT ferrite, respectively. Compression axis is along the vertical direction of the map.

Supplementary Table S1 Chemical composition of the alloy studied (mass%).

C	Ni	Mn	P	Si	Al	S	Fe
0.111	10.08	0.01	0.001	0.006	0.33	0.0017	Bal.
